# Identification and validation of a 44-gene expression signature for the classification of renal cell carcinomas

**DOI:** 10.1186/s13046-017-0651-9

**Published:** 2017-12-06

**Authors:** Qifeng Wang, Hualei Gan, Chengshu Chen, Yifeng Sun, Jinying Chen, Midie Xu, Weiwei Weng, Liyu Cao, Qinghua Xu, Jian Wang

**Affiliations:** 1 Department of Pathology, Fudan University Shanghai Cancer Center, Shanghai, China; 2 Department of Oncology, Shanghai Medical College, Fudan University, Shanghai, China; 3 Canhelp Genomics, Hangzhou, Zhejiang China; 4 Institute of Machine Learning and Systems Biology, College of Electronics and Information Engineering, Tongji University, Shanghai, China; 5 Department of Biomedical Engineering, University of California, Irvine, USA

**Keywords:** Renal cell carcinomas, Gene expression profiling, Microarray, Next-generation sequencing, Quantitative real-time PCR

## Abstract

**Background:**

Renal cancers account for more than 3% of all adult malignancies and cause more than 23,400 deaths per year in China alone. The four most common types of kidney tumours include clear cell, papillary, chromophobe and benign oncocytoma. These histological subtypes vary in their clinical course and prognosis, and different clinical strategies have been developed for their management. Some kidney tumours can be very difficult to distinguish based on the pathological assessment of morphology and immunohistochemistry.

**Methods:**

Six renal cell carcinoma microarray data sets, including 106 clear cell, 66 papillary, 42 chromophobe, 46 oncocytoma and 35 adjacent normal tissue samples, were subjected to integrative analysis. These data were combined and used as a training set for candidate gene expression signature identification. In addition, two independent cohorts of 1020 RNA-Seq samples from *The Cancer Genome Atlas* database and 129 qRT-PCR samples from Fudan University Shanghai Cancer Center (FUSCC) were analysed to validate the selected gene expression signature.

**Results:**

A 44-gene expression signature derived from microarray analysis was strongly associated with the histological differentiation of renal tumours and could be used for tumour subtype classification. The signature performance was further validated in 1020 RNA-Seq samples and 129 qRT-PCR samples with overall accuracies of 93.4 and 93.0%, respectively.

**Conclusions:**

A 44-gene expression signature that could accurately discriminate renal tumour subtypes was identified in this study. Our results may prompt further development of this gene expression signature into a molecular assay amenable to routine clinical practice.

## Background

According to the newest Globocan 2012, renal cancers are the 17th most common malignancy, accounting for more than 3% of adult malignancies and causing approximately 23,400 deaths per year in China alone [1, 2]. In 2011, the overall incidence of renal cancers in China rose to 3.35 cases per 10^5^ people, and the estimated mortality rate was 1.12 deaths per 10^5^ people [3]. According to the 2016 World Health Organization (WHO) classification, there are 16 subtypes of renal cell carcinoma (RCC), a family of carcinomas that arise from renal tubule epithelia [4]. Currently, the four most common types of kidney tumours include clear cell RCC (ccRCC), papillary RCC (pRCC), chromophobe RCC (chRCC) and benign oncocytoma [4]. These histological subtypes vary in their clinical course and outcomes, and different clinical management strategies have been developed for their treatment. Among patients with the four most common types, patients with ccRCC have the worst prognosis, and there are differences between the prognosis of patients with pRCC and chRCC [5]. Different genetic alterations induce the development of renal tubules into RCCs of varying histological subtypes that exhibit different gene expression patterns or mutations, thus providing specific molecular candidates for targeted therapy (e.g., mTOR, VEGF, KIT, and checkpoint inhibitors) [6]. Improving the molecular understanding of the mechanisms underlying RCC subtypes has facilitated the development of targeted therapies and biomarkers in response to treatment [6]. Distinguishing between some types of kidney tumours based on morphology and immunohistochemistry can be very difficult for pathologists, while the correct identification of these subtypes is important for making precise decisions regarding therapeutic regimens.

Recent studies focused on microarray profiling of different RCC subtypes to develop accurate diagnostic RCC biomarkers. Using microarray analysis of renal tumours, claudin-7 mRNA, a distal nephron marker, was overexpressed in chRCC compared with that in oncocytoma, ccRCC, and pRCC [7]. Further immunohistochemical analysis of two independent cohorts showed that claudin-7 expression was detected in 67 and 100% of chRCCs, 0 and 7% of ccRCCs, 28 and 90% of pRCCs, and 26 and 45% of oncocytomas [8, 9]. These studies revealed the potential of claudin-7 as a biomarker for distinguishing chRCC from the remaining three RCC subtypes and indicated the accuracy of microarray technology for detecting diagnostic biomarkers. Compared with classifying diseases using a single gene marker, simultaneously quantifying the expression of numerous genes may potentially capture the complex physiopathology underlying tumourigenesis and the development of specific RCC subtypes. Several studies have used microarray technology to identify gene expression signatures for the classification of RCCs. Chen and coworkers published a four-gene panel that could classify RCC subtypes with an estimated prediction accuracy of 96% [10]. Youssef and colleagues also reported a classification system using miRNA signatures with a maximum of four steps that had sensitivities of 97% for distinguishing normal cells from RCC, 100% for the ccRCC subtype, 97% for the pRCC subtype, and 100% accuracy in distinguishing the oncocytoma subtype from the chRCC subtype [11].

In this study, to identify novel gene biomarkers for the classification of RCC subtypes, we performed an integrative analysis of six microarray data sets (*n* = 295). The selected genes in the training set were validated in 1020 RNA-sequencing samples from *The Cancer Genome Atlas* (*TCGA*) database and then tested in 129 independent specimens by qRT-PCR. A 44-gene signature was identified and validated as being highly sensitive and specific for the classification of RCCs.

## Methods

### Gene expression database curation

Gene expression data sets of 1315 renal tumours with histologically confirmed subtypes and adjacent normal tissues were collected from public data repositories (e.g., ArrayExpress, Gene Expression Omnibus (GEO), and *TCGA* data portal) and curated to form a comprehensive RCC transcriptome database. Array-based gene expression profiling of 295 tissue samples obtained from six GEO data sets (GSE12090, GSE15641, GSE19949, GSE8271, GSE7023 and GSE19982) was mainly conducted on two different Affymetrix oligonucleotide microarray platforms, GeneChip Human Genome U133A Array and U133Plus 2.0 Array. Detailed descriptions of the specimen characteristics and clinical features are provided in the original studies [12–15]. The sequence-based gene expression profiles of 1020 tissue samples (including 534 ccRCC, 291 pRCC, 66 chRCC and 129 normal kidney samples) were generated on an Illumina HiSeq 2000 RNA sequencing platform and retrieved from the cBioPortal for Cancer Genomics [16]. The gene expression profiles consisted of transcriptomic data for 20,500 unique genes, and clinical information for the selected samples was retrieved from the “Clinical Biotab” section of the data matrix based on the Biospecimen Core Resource IDs of the patients.

### Microarray data processing and normalization

Gene expression data analysis was performed using R software and packages from the Bioconductor project [17–19]. We used the Single Channel Array Normalization (SCAN) approach from the SCAN-UPC package to process Affymetrix microarray data [20, 21]. Upon normalising each raw CEL file, SCAN outputs probe-level expression values. We further used the custom mapping files from the BrainArray resource to summarise probe-level intensities directly to gene-level expression values [22]. Thus, probes mapping to multiple genes and other problems associated with older generations of Affymetrix probe designs were avoided. After normalization, we applied the ComBat approach to adjust for batch effects [23].

### Gene signature identification and performance assessment

To identify a gene expression signature, we used the support vector machine-recursive feature elimination (SVM-RFE) algorithm for feature selection and classification modelling [24]. For multi-class classification, a one-versus-all approach was used by which multiple binary classifiers were first derived for each subtype. The results are reported as the subtype classifying the test sample with the highest confidence. For each specimen, the predicted subtype was compared with the reference diagnosis, and a true positive result was indicated when the predicted subtype matched the reference diagnosis. When the predicted subtype and reference diagnosis did not match, the specimen was considered a false positive. For each subtype on the panel, sensitivity was defined as the ratio of true positive results to the total positive samples analysed, while specificity was defined as the ratio (1 - false positive)/(total tested - total positive).

### Biological network and functional enrichment analysis

Enrichment analysis of Gene Ontology and molecular pathways was performed using the Lynx Systems Biology Tool [25]. All significance tests were two-sided, and a false discovery rate less than 0.05 was considered significant. Biological network analysis was performed with NetworkAnalyst software [26, 27]. Protein-protein interaction information was retrieved from the IMEx Interactome Database [28]. A dense network was connected by retaining only the seed proteins as well as minimum essential non-seed proteins to study the key interactions.

### qRT-PCR analysis

We included 121 renal tumour samples and 8 non-tumour kidney tissues for qRT-PCR analyses. Written informed consent was obtained from all participants. The study was approved by the Ethics Committee of Fudan University Shanghai Cancer Center (FUSCC), China. Of the 121 tumours, 26 were ccRCC, 40 were chRCC, 28 were pRCC, and 27 were oncocytoma. Total RNA was isolated from formalin-fixed paraffin-embedded (FFPE) tissue sections using a FFPE Total RNA Isolation Kit (Canhelp Genomics, Hangzhou, China). Briefly, the paraffin sections were placed in sterile 1.5-ml microcentrifuge tubes, deparaffinized with 100% xylene, and washed twice with 100% ethanol. The deparaffinized tissue was digested with proteinase K at 56 °C for 15 min and then incubated at 80 °C for another 15 min to partially reverse nucleic acid crosslinking. The samples were treated with DNase and eluted in 40 μl RNase-free water. The concentration of total RNA was spectrophotometrically determined using total absorbance at 260 nm, and the purity was quantified using the A260/A280 ratio. RNA samples with A260/A280 ratios of 1.9 ± 0.2 were included in this study.

For each sample, cDNA was generated from isolated total RNA using a High-Capacity cDNA Reverse Transcription Kit with RNase Inhibitor (Applied Biosystems, Foster City, CA, Unites States). Primers and MGB probes for the tested gene candidates and control gene were designed using Primer Express software (Applied Biosystems). Subsequently, the expression level of gene candidates was analysed on an Applied Biosystems 7500 Real-Time PCR system using TaqMan Gene Expression Assays (Applied Biosystems). The PCR program was initiated at 95 °C for 10 min, followed by 40 thermal cycles, each at 95 °C for 15 s and at 60 °C for 1 min.

## Results

### Establishment of the RCC Transcriptome database

**Fig. 1:**
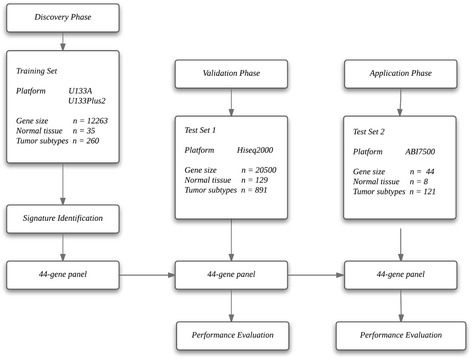
Flow diagram of gene expression signature identification and performance assessment. Gene expression profiles were retrieved from the U133A/ U133APlus2 microarray through a bioinformatics approach. Gene expression analyses were performed in the training set (six GEO microarray datasets) first and then validated in TCGA cohort two validation sets (and the FUSCC cohort)

To create a RCC transcriptome database for subtype classification, we performed a systematic search of major biological data repositories (e.g., ArrayExpress, GEO, and *TCGA*) to collect gene expression data sets from ccRCC, pRCC, chRCC, oncocytoma and adjacent normal tissue samples. Overall, we accumulated the gene expression profiles of 1315 tissue samples to form a comprehensive RCC transcriptome database. To identify a reliable gene expression signature, we adopted a training-testing-validation approach in this study. First, the microarray-based gene expression profiles of 295 specimens were retrieved from the database and curated to form a training set. Second, two independent sets were used to test and validate the classification performance of the gene expression-based signature; one was composed of the sequence-based gene expression profiles of 1020 specimens (Test Set 1), and the other was composed of the gene expression profiles of 129 specimens that were analysed with qRT-PCR (Test Set 2). Figure 1 depicts the three distinct phases of our study design, and Table 1 summarises the clinical characteristics of the samples in the study.

**Table 1:** Summary of sample information

Samples	Training set		Test Set 1		Test Set 2	
	n	%	n	%	n	%
Normal tissue	35	11.9	129	12.6	8	6.2
RCC subtypes						
ccRCC	106	35.9	534	52.4	26	20.2
chRCC	42	14.2	66	6.5	40	31
pRCC	66	22.4	291	28.5	28	21.7
Oncocytoma	46	15.6	0	0	27	20.9
Total	295	100	1020	100	129	100

### Identification of a 44-gene signature in the training set

**Fig. 2:**
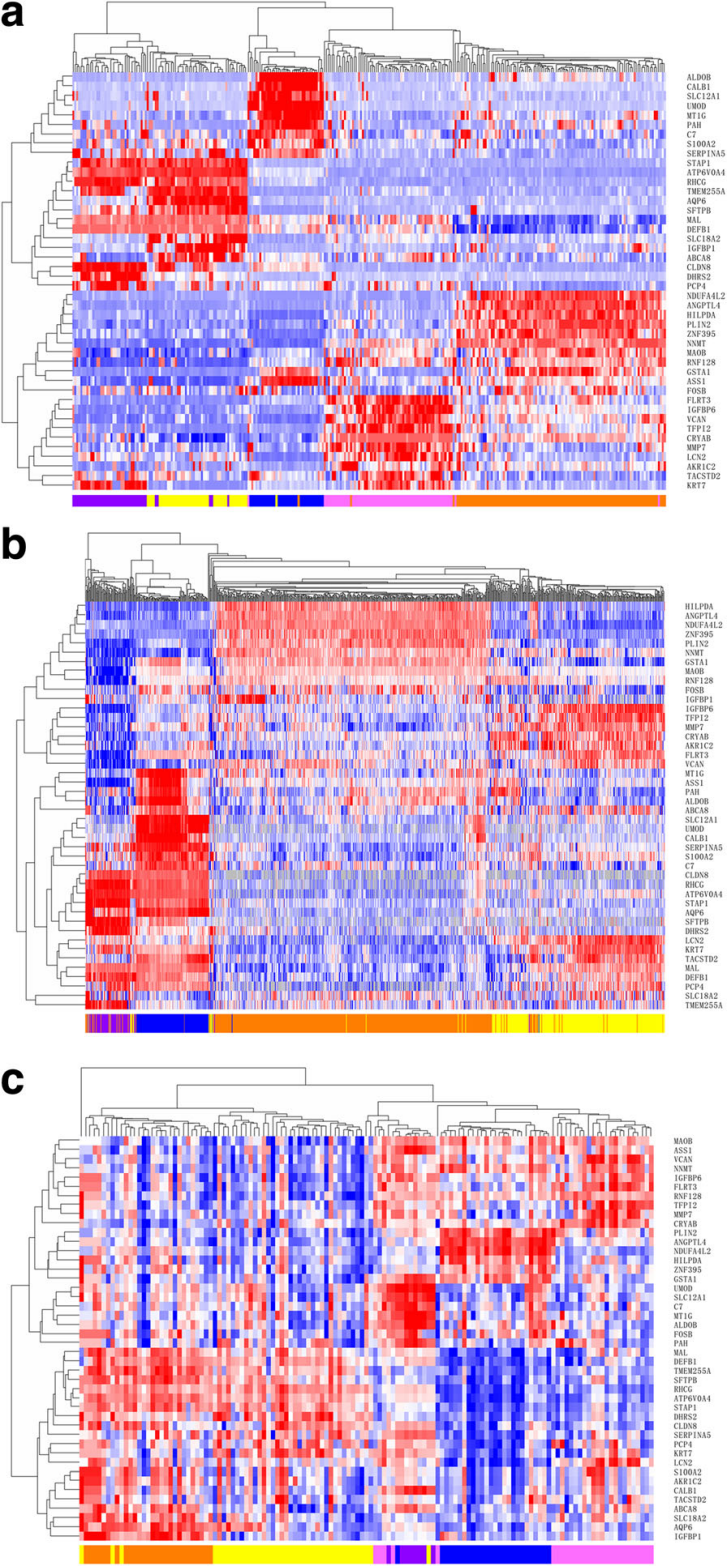
Hierarchical clustering analysis of 44-gene expression data in the training set and test sets. **a** Hierarchical clustering of 295 samples from the training set. Normalized gene expression intensities were shifted to mean = 0, and rescaled to STD = 1 to enhance the expression differences. The average linkage hierarchical clustering method was performed where the metric of similarity was Pearson’s correlation between every pair of samples. The right panel indicates the official symbol of 44 genes. The left panel shows a dendrogram of hierarchical clustering of these genes. Colored pixels capture the magnitude of the expression for any gene, where shades of red and blue represent over-expression and under-expression, respectively, relative to the mean for each gene. The upper panel shows a dendrogram of hierarchical clustering of samples. The histological type of each sample is indicated in the bottom panel, with chromophobe tumours shown in purple, clear cell tumours shown in orange, oncocytoma samples indicated in yellow, papillary tumours in pink, and adjacent tissue samples in blue. The samples clustered into five groups that closely follow the histological types. Among the four tumour subtypes, the oncocytoma and chromophobe samples cluster together, whereas the conventional tumours show a higher degree of similarity to papillary tumours. **b** Hierarchical clustering of 1020 samples from the Test Set 1. **c** Hierarchical clustering of 129 samples from the Test Set 2

The training set consisted of 106 ccRCC, 66 pRCC, 42 chRCC, 46 oncocytoma and 35 adjacent normal tissue samples. After the data normalization and annotation steps, a matrix of 12,263 unique genes in 295 samples (≈ 3.5 million data points) was prepared for downstream bioinformatics analyses. Extracting a subset of informative genes from high-dimension genomic data is a critical step for gene expression signature identification. Although many algorithms have been developed, the SVM-RFE approach is considered one of the best gene selection algorithms. For each subtype, we used the SVM-RFE approach to (1) evaluate and rank the contributions of each gene to the optimal separation of a specific subtype from other subtypes; (2) select the top 10 ranked genes as the most differentially expressed for that subtype; (3) repeat the process for each subtype, and obtain 5 lists of the top 10 gene set. After removing redundant features, 44 unique genes (listed in Table 2) were obtained and used to cluster the 295 training set samples. The average linkage hierarchical clustering method was performed where the metric of similarity was Pearson’s correlation between the 44-gene expression profiles of the samples. As shown in Fig. 2a, the samples were clustered into five groups that closely followed the histological subtypes. Among the four tumour subtypes, the oncocytoma and chRCC samples clustered together, whereas the ccRCC samples were more similar to pRCC samples.

**Table 2:** Descripotion of 44 genes annotation

Gene Symbol	Gene description	Cytoband
ABCA8	ATP-binding cassette, sub-family A (ABC1), member 8	17q24
AKR1C2	aldo-keto reductase family 1, member C2	10p15-p14
ALDOB	aldolase B, fructose-bisphosphate	9q21.3-q22.2
ANGPTL4	angiopoietin-like 4	19p13.3
AQP6	aquaporin 6, kidney specific	12q13
ASS1	argininosuccinate synthase 1	9q34.1
ATP6V0A4	ATPase, H+ transporting, lysosomal V0 subunit a4	7q34
C7	complement component 7	5p13
CALB1	calbindin 1, 28 kDa	8q21.3
CLDN8	claudin 8	21q22.11
CRYAB	crystallin, alpha B	11q22.3-q23.1
DEFB1	defensin, beta 1	8p23.1
DHRS2	dehydrogenase/reductase (SDR family) member 2	14q11.2
FLRT3	fibronectin leucine rich transmembrane protein 3	20p11
FOSB	FBJ murine osteosarcoma viral oncogene homolog B	19q13.32
GSTA1	glutathione S-transferase alpha 1	6p12.1
HILPDA	hypoxia inducible lipid droplet-associated	7q32.1
IGFBP1	insulin-like growth factor binding protein 1	7p12.3
IGFBP6	insulin-like growth factor binding protein 6	12q13
KRT7	keratin 7, type II	12q13.13
LCN2	lipocalin 2	9q34
MAL	mal, T-cell differentiation protein	2q11.1
MAOB	monoamine oxidase B	Xp11.23
MMP7	matrix metallopeptidase 7 (matrilysin, uterine)	11q21-q22
MT1G	metallothionein 1G	16q13
NDUFA4L2	NADH dehydrogenase (ubiquinone) 1 alpha subcomplex, 4-like 2	12q13.3
NNMT	nicotinamide N-methyltransferase	11q23.1
PAH	phenylalanine hydroxylase	12q22-q24.2
PCP4	Purkinje cell protein 4	21q22.2
PLIN2	perilipin 2	9p22.1
RHCG	Rh family, C glycoprotein	15q25
RNF128	ring finger protein 128, E3 ubiquitin protein ligase	Xq22.3
S100A2	S100 calcium binding protein A2	1q21
SERPINA5	serpin peptidase inhibitor, clade A (alpha-1 antiproteinase, antitrypsin), member 5	14q32.1
SFTPB	surfactant protein B	2p12-p11.2
SLC12A1	solute carrier family 12 (sodium/potassium/chloride transporter), member 1	15q15-q21.1
SLC18A2	solute carrier family 18 (vesicular monoamine transporter), member 2	10q25
STAP1	signal transducing adaptor family member 1	4q13.2
TACSTD2	tumor-associated calcium signal transducer 2	1p32
TFPI2	tissue factor pathway inhibitor 2	7q22
TMEM255A	transmembrane protein 255A	Xq24
UMOD	uromodulin	16p12.3
VCAN	versican	5q14.3
ZNF395	zinc finger protein 395	8p21.1

### Functional enrichment and biological network analysis

**Fig. 3:**
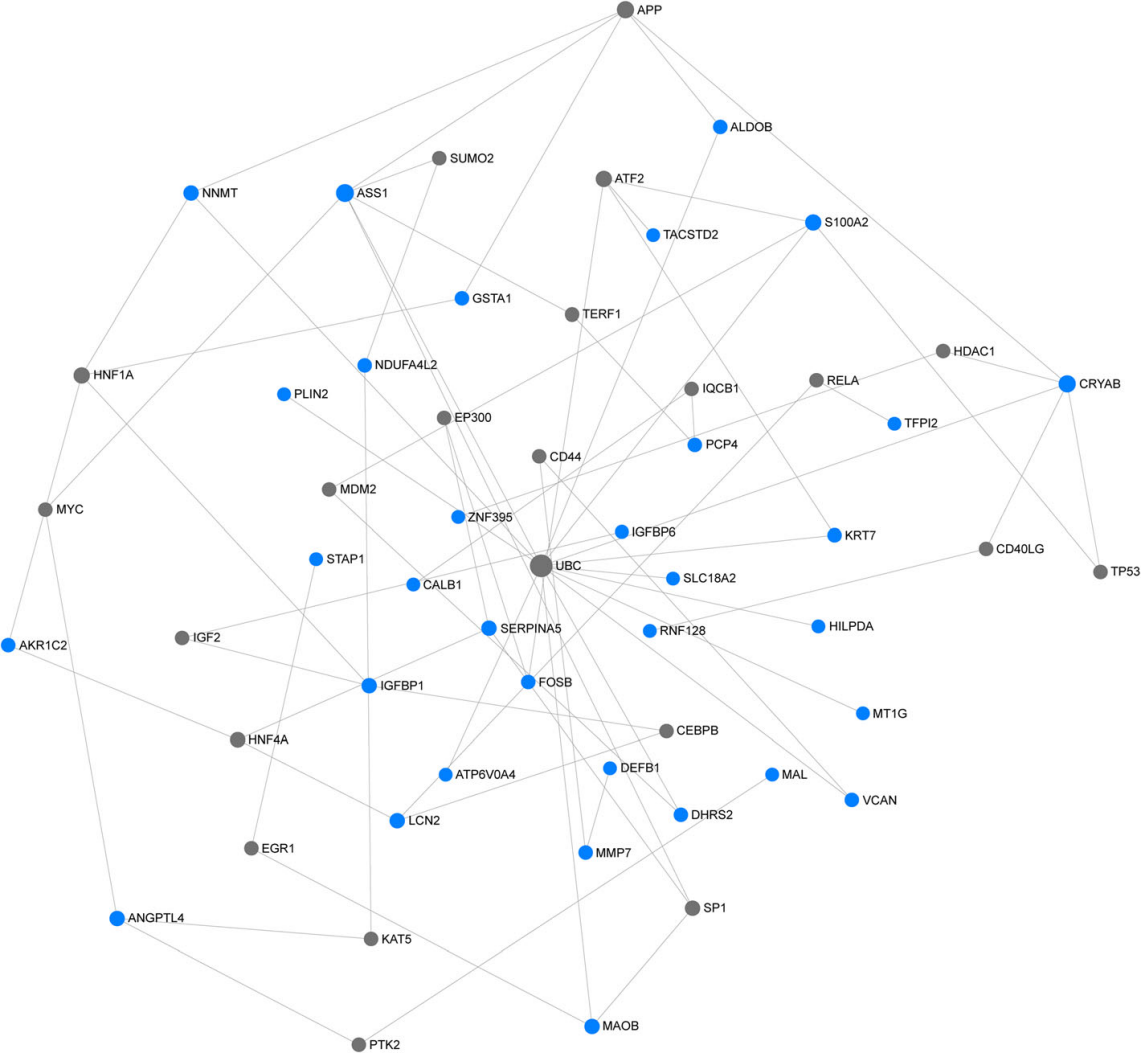
Protein-protein interaction network of the 44-gene set. The network of protein-protein interactions of the 44-gene set inferred using the IMEx Interactome Database. Blue nodes indicate the proteins present in the 44-gene set, whereas grey nodes represent proteins not in the 44-gene set. The size of the node is proportional to the degree of connections. The large nodes represent a few high-degree hub nodes, while most small nodes have only a few connections

We further investigated whether the 44 candidate genes exhibited biological features relevant to renal carcinogenesis. As shown in Table 3, the most significantly enriched gene categories are involved in insulin-like growth factor binding, transmembrane transport of small molecules, cocaine, amphetamine addiction, etc. Interestingly, seven of the 44 candidate genes (ASS1, DEFB1, IGFBP6, LCN2, SERPINA5, UMOD and VCAN) were indeed overrepresented in the “Renal-cell cancer” gene set (*p* < 1.4 E-5). More specifically, AQP6, CLDN8 and KRT7 were overrepresented in the “Renal oncocytoma” gene set (*p* < 6.1 E-6). We also explored the underlying biological networks of these 44 candidate genes. We used the 44 genes as seeds to generate a minimum protein-protein interaction network. As shown in Fig. 3, the network includes 33 genes of the 44-gene set and is centred on essential nodes such as APP, ASS1, ATF2, CRYAB, HNF1A, S100A2 and UBC. Enrichment analysis revealed that the most significant molecular networks were the TGF beta signalling pathway, Androgen receptor signalling pathway, Transcriptional misregulation in cancer, etc. (Table 4).

**Table 3:** GO and KEGG pathway analysis of 44 gene

Data source	Feature ID	Name	Genes	*P* Value
GO Molecular Function	GO:0031995	insulin-like growth factor II binding	IGFBP1, IGFBP6	1.25E-04
GO Molecular Function	GO:0031994	insulin-like growth factor I binding	IGFBP1, IGFBP6	1.60E-04
GO Molecular Function	GO:0005539	glycosaminoglycan binding	SERPINA5, VCAN	1.00E-03
GO Molecular Function	GO:0016597	amino acid binding	ASS1, PAH	1.00E-03
GO Biological Process	GO:0007588	excretion	AQP6, ATP6V0A4, SLC12A1, UMOD	1.76E-06
GO Biological Process	GO:0071242	cellular response to ammonium ion	ASS1, SLC18A2	4.50E-06
GO Biological Process	GO:0048878	chemical homeostasis	SLC12A1, UMOD	1.35E-05
GO Biological Process	GO:0051412	response to corticosterone	FOSB, MAOB, SLC18A2	1.38E-05
GO Cellular Component	GO:0005615	extracellular space	ANGPTL4, DEFB1, FLRT3, HILPDA, IGFBP1, IGFBP6, LCN2, MMP7, SERPINA5, SFTPB, TACSTD2, UMOD, VCAN	8.42E-07
GO Cellular Component	GO:0016324	apical plasma membrane	AQP6, ATP6V0A4, MAL, RHCG, SLC12A1, UMOD	2.02E-05
GO Cellular Component	GO:0005576	extracellular region	ANGPTL4, C7, DEFB1, IGFBP1, IGFBP6, LCN2, MMP7, PLIN2, SERPINA5, TFPI2, UMOD, VCAN	3.77E-05
GO Cellular Component	GO:0005578	proteinaceous extracellular matrix	ANGPTL4, FLRT3, MMP7, TFPI2, VCAN	1.43E-04
Cancer Gene Index [CGI]	C4863	prostate cancer	AKR1C2, ANGPTL4, ASS1, GSTA1, IGFBP1, IGFBP6, LCN2, MAL, MT1G, S100A2, TFPI2, VCAN	3.20E-06
DISEASE DB (Univ of Copenhagen)	DOID:6245	Renal oncocytoma	AQP6, CLDN8, KRT7	6.14E-06
Cancer Gene Index [CGI]	C9385	renal-cell cancer	ASS1, DEFB1, IGFBP6, LCN2, SERPINA5, UMOD, VCAN	1.41E-05
Cancer Gene Index [CGI]	C2978	cysts	GSTA1, IGFBP1, IGFBP6, LCN2, MAL, UMOD	1.45E-05
KEGG	path:hsa05030	Cocaine addiction	FOSB, MAOB, SLC18A2	1.68E-04
REACTOME	REACT_15518	Transmembrane transport of small molecules	ABCA8, AQP6, ATP6V0A4, LCN2, RHCG, SLC12A1, SLC18A2	2.28E-04
KEGG	path:hsa05031	Amphetamine addiction	FOSB, MAOB, SLC18A2	4.19E-04
KEGG	path:hsa01230	Biosynthesis of amino acids	ALDOB, ASS1, PAH	1.00E-03
KEGG	path:hsa00360	Phenylalanine metabolism	MAOB, PAH	1.00E-03

**Table 4:** Top 20 enriched pathways of 55 genes within network

Feature ID	Name	Data source	Genes	*P* Value
WP366	TGF beta Signaling Pathway	WIKIPATHWAYS	APP ATF2 EP300 FOSB HDAC1 MYC PTK2 SP1 TP53	4.97E-07
WP138	Androgen receptor signaling pathway	WIKIPATHWAYS	EP300 HDAC1 KAT5 MDM2 PTK2 RELA SP1	4.27E-06
path:hsa05202	Transcriptional misregulation in cancer	KEGG	CEBPB HDAC1 MDM2 MYC PTK2 RELA SP1 TP53	2.02E-05
WP2377	Integrated Pancreatic Cancer Pathway	WIKIPATHWAYS	APP EGR1 EP300 HNF4A MDM2 MYC SP1 TP53	3.49E-05
path:hsa05169	Epstein-Barr virus infection	KEGG	ATF2 CD44 EP300 HDAC1 MDM2 MYC RELA TP53	4.12E-05
path:hsa05030	Cocaine addiction	KEGG	ATF2 FOSB MAOB RELA SLC18A2	1.33E-04
REACT_169274	Cellular Senescence	REACTOME	CEBPB MDM2 RELA SP1 TERF1 TP53 UBC	3.43E-04
path:hsa05031	Amphetamine addiction	KEGG	ATF2 FOSB HDAC1 MAOB SLC18A2	4.35E-04
REACT_169325	Oncogene Induced Senescence	REACTOME	MDM2 SP1 TP53 UBC	4.68E-04
REACT_120734	SMAD2/SMAD3:SMAD4 heterotrimer regulates transcription	REACTOME	HDAC1 MYC SP1 UBC	1.00E-03
path:hsa05220	Chronic myeloid leukemia	KEGG	HDAC1 MDM2 MYC RELA TP53	1.00E-03
REACT_120956	Cellular responses to stress	REACTOME	CEBPB EP300 MDM2 RELA SP1 TERF1 TP53 UBC	1.00E-03
WP1984	Integrated Breast Cancer Pathway	WIKIPATHWAYS	EP300 HDAC1 MDM2 MYC SP1 TP53	1.00E-03
WP254	Apoptosis	WIKIPATHWAYS	IGF2 MDM2 MYC RELA TP53	1.00E-03
REACT_121061	Transcriptional activity of SMAD2/SMAD3:SMAD4 heterotrimer	REACTOME	HDAC1 MYC SP1 UBC	1.00E-03
path:hsa05166	HTLV-I infection	KEGG	ATF2 EGR1 EP300 KAT5 MYC RELA TP53	2.00E-03
WP399	Wnt Signaling Pathway and Pluripotency	WIKIPATHWAYS	CD44 EP300 MMP7 MYC TP53	2.00E-03
REACT_118780	NOTCH1 Intracellular Domain Regulates Transcription	REACTOME	EP300 HDAC1 MYC UBC	2.00E-03
REACT_299	Signaling by NOTCH	REACTOME	EP300 HDAC1 MYC TP53 UBC	3.00E-03
h_arfPathway	Tumor Suppressor Arf Inhibits Ribosomal Biogenesis	BIOCARTA	MDM2 MYC TP53	3.00E-03

### Performance assessment with 5-fold cross-validation

As an initial step, we assessed the performance of the classifier using 5-fold cross-validation within the training set. In 5-fold cross-validation, we created the training and testing sets by splitting the data into five equally sized subsets. We treated a single subsample as the testing set and the remaining data as the training set. We then ran and tested models on all five datasets and averaged the estimates. Given the limited sample size of the training set, we repeated the 5-fold cross-validation process 1000 times and estimated the average classification accuracy and corresponding 95% confidence interval (95% CI). The 44-gene expression signature showed an overall accuracy of 95.7% (95% CI: 0.912 to 1.00) with notable variation between different subtypes. Sensitivities ranged from 88.0% (chRCC) to 98.1% (ccRCC). Using this internal validation of the training set, these data provided a preliminary estimate of classification performance.

### Independent validation in renal Tumours profiled with next-generation sequencing

The final classification model of the 44-gene expression signature was established using the entire training set and then applied to an independent validation set comprising 534 ccRCC, 291 pRCC, 66 chRCC and 129 adjacent normal tissue specimens profiled with next-generation sequencing (Test Set 1). The hierarchical clustering of 44 genes and 1020 samples revealed distinct patterns between ccRCC, pRCC, chRCC and adjacent normal samples (Fig. 2b). With the 44-gene expression signature, 524 samples were classified as ccRCC, 284 as pRCC, 81 as chRCC, 9 as oncocytoma and 122 as normal kidney tissues. Overall, the gene expression-based assignments reached a 93.4% overall agreement with the reference diagnoses (953 of 1020; 95% CI: 0.917 to 0.948). Sensitivities ranged from 90.9% (chRCC) to 94.6% (normal tissue), while specificities ranged from 95.7% (chRCC) to 100% (normal tissue). The detailed sensitivities and specificities are listed in Table 5.

**Table 5:** Performance characteristics of the 44-gene expression signature in two test sets

	Test Set 1			Test Set 2		
	n	Sensitivity	Specificity	n	Sensitivity	Specificity
Normal	129	94.6%	100.0%	8	100.0%	98.3%
ccRCC	534	94.2%	95.7%	26	96.2%	96.1%
chRCC	66	90.9%	97.8%	40	92.5%	97.8%
pRCC	291	92.1%	97.8%	28	89.3%	99.0%
Oncocytoma	/	/	/	27	92.6%	98.2%
Total	1020	Overall accuracy = 93.4%		129	Overall accuracy = 93.0%	

### Clinical validation of the 44-gene signature by qRT-PCR analysis

Microarray and RNA-sequencing data provide a global assessment of transcriptomic variations, but their resolution and accuracy are limited in individual gene analyses, and they remain difficult to use in clinical practice. qRT-PCR is generally considered the “standard procedure” assay for measuring individual gene expression and often used to confirm the findings of microarray and RNA-sequencing analyses. Hence, we further evaluated the expression levels of 44 genes by qRT-PCR in an independent cohort of 121 RCC tumours (comprising 26 ccRCC, 28 pRCC, 40 chRCC, and 27 oncocytoma) and 8 normal kidney tissues (Test Set 2). Figure 2c shows the hierarchical clustering of the 44 genes and 129 samples based on the qRT-PCR data. As seen in the figure, distinct patterns were observed between four tumour subtypes and adjacent normal samples. With the 44-gene expression signature, 29 samples were classified as ccRCC, 25 as pRCC, 39 as chRCC, 26 as oncocytoma and 10 as normal kidney tissues. Overall, the gene expression-based assignments reached 93.0% overall agreement with the reference diagnoses (120 of 129; 95% CI: 0.868 to 0.966). Sensitivities ranged from 89.3% (pRCC) to 100% (normal tissue), while specificities ranged from 96.1% (ccRCC) to 100% (chRCC and pRCC). The detailed sensitivities and specificities are listed in Table 5.

## Discussion

Due to the comprehensive development of high-throughput microarray and next-generation sequencing technologies, as well as the comprehensive efforts of systematic cancer genomics projects, numerous genomic data sets were utilised in our research. In this study, we identified a 44-gene expression signature for the accurate and robust classification of RCC subtypes (ccRCC, pRCC, chRCC, and oncocytoma). The 44-gene expression signature demonstrated an overall accuracy of 95.7% for 4 RCC subtypes by cross-validation of the training set profiled with the high-throughput microarray and 93.4% in an independent test set of 1020 RCC and normal kidney samples profiled with next-generation sequencing. Furthermore, we tested the signature on an independent cohort by qRT-PCR. An overall accuracy of 93.0% was achieved with the 129 RCC samples with 4 subtypes and normal specimens. This signature may serve as a reliable diagnostic tool to aid pathologists with the growing unmet need for RCC classification.

Kidney tumour subtypes are characterised by different genetic mutations and chromosomal variations and thus present different gene expression profiles. Numerous molecules have been reported as capable of distinguishing kidney tumour subtypes. For example, vascular cell adhesion molecule 1 (VCAM1) was reportedly significantly up-regulated in ccRCC and pRCC, whereas it was down-regulated in chRCC and oncocytoma [29]. Furthermore, positive immunoreactivity of the metastasis suppressor protein KAI1 was often detected in chRCC specimens and rarely in ccRCC and oncocytoma specimens [30], and GST-alpha mRNA expression was higher in most ccRCCs than in other kidney tumours [31]. However, in addition to being unable to consistently distinguish RCC subtypes based on regular microscopic morphology, single molecules seldom exhibit extensive power for classifying all 4 major renal tumour subtypes. Therefore, comprehensive analysis of multiple gene expression panels is necessary for the classification of renal tumour types.

Based on the expression patterns of 44 genes, we classified the 4 most common renal tumour subtypes, ccRCC, pRCC, chRCC, and oncocytoma, with sensitivities ranging from 88% (chRCC) to 98% (ccRCC) in the training set, 90.9% (chRCC) to 94.6% (normal tissue) in Test Set 1, and 89.3% (pRCC) to 100% (normal tissue) in Test Set 2. In addition, the diagnostic histological classification accuracy was higher than that obtained with any of the genes used alone. The chRCC and oncocytoma samples displayed almost identical gene expression profiles for MAL, TMEM255A, RHCG, ATP6V0A4, STAP1, and DEFB1, as demonstrated by both RNA microarray and RNA sequencing, which is in agreement with the known fact that chRCC and oncocytoma are related neoplasms [32]. However, because chRCC is potentially malignant, and oncocytoma appears to be a benign mimic of RCC [4, 33], the potential subtle difference in gene expression is expected, and the distinction between both subtypes has important clinical significance. Thus, we proposed that biomarkers identified by gene expression profiles accumulated from large cohorts indeed help to discriminate important and difficult differential diagnoses.

Several studies have reported the promise of gene or protein expression-based signatures in the classification of RCC subtypes. Unlike many studies in which samples were often collected from single central or ethnic cohorts, our approach exploited tumour samples from two large databases; samples extracted from the GEO database were used for construction of the classification panel, and samples from the *TCGA* database were extracted for testing our 44-gene expression signature. In addition, we further validated our 44-gene expression signature in an independent Chinese cohort using qRT-PCR. In a clinical scenario, the application of multi-centre, multi-ethnic data would greatly increase the reliability and universal applicability of our 44-gene expression signature. In this study, we showed that the 44-gene expression signature could reliably identify the tumour subtypes in 95.7% of the 295 samples tested. This accuracy is comparable to that of other signatures established by mRNA or miRNA biomarkers (ranging from 90 to 96%) [10, 11, 34]. The performance of this mRNA signature analysis by qRT-PCR also compares favourably with protein signature analysis by immunohistochemistry, the current clinical practice standard, which has shown 78–87% accuracy in identifying RCC samples using AMACR, CK7, and CD10 [35]. Moreover, analysis of the expression patterns of 44 genes by qRT-PCR classified the 4 most common renal tumour subtypes with 100% sensitivity in distinguishing normal from RCC, 96.2% for the ccRCC subtype, 92.5% for the chRCC subtype, 89.3% for the pRCC subtype, and 92.6% for the oncocytoma subtype; this signature is also comparable to other signatures (97% in distinguishing normal from RCC, 98%–100% for the ccRCC subtype, 93% for the chRCC subtype, 97–98% for the pRCC subtype, and 86% for the oncocytoma subtype) [10, 11, 34].

In routine clinical settings, the most commonly used diagnostic materials are FFPE samples; thus, further research is needed to successfully translate the 44-gene signature from gene expression microarrays and qRT-PCR to immunohistochemistry, thus allowing widespread access and applications in clinical diagnoses.

## Conclusion

In conclusion, in the present study, we developed and validated a 44-gene expression-based signature for the classification of RCC subtypes. Our results may prompt further development of this gene expression signature into a molecular assay amenable to routine clinical practice. We foresee its application in cases wherein morphology and immunohistochemistry fail to distinguish between renal tumour subtypes. Further studies are needed to determine the role of our gene expression-based signature in personalised therapy choices and the prognosis of therapeutic outcomes for RCC patients with different subtypes.
